# Supplementary material for: Renal Corin Is Essential for Normal Blood Pressure and Sodium Homeostasis
Source: Int J Mol Sci. 2022 Sep 24;23(19):11251. doi: 10.3390/ijms231911251 (PMC9570390; doi:10.3390/ijms231911251)
Supplement: Supplementary file 1 [file ijms-23-11251-s001.zip › ijms-1899585-SI.pdf]

Renal Corin Is Essential for Normal Blood Pressure and Sodium Homeostasis

Supplementary Figures and Table

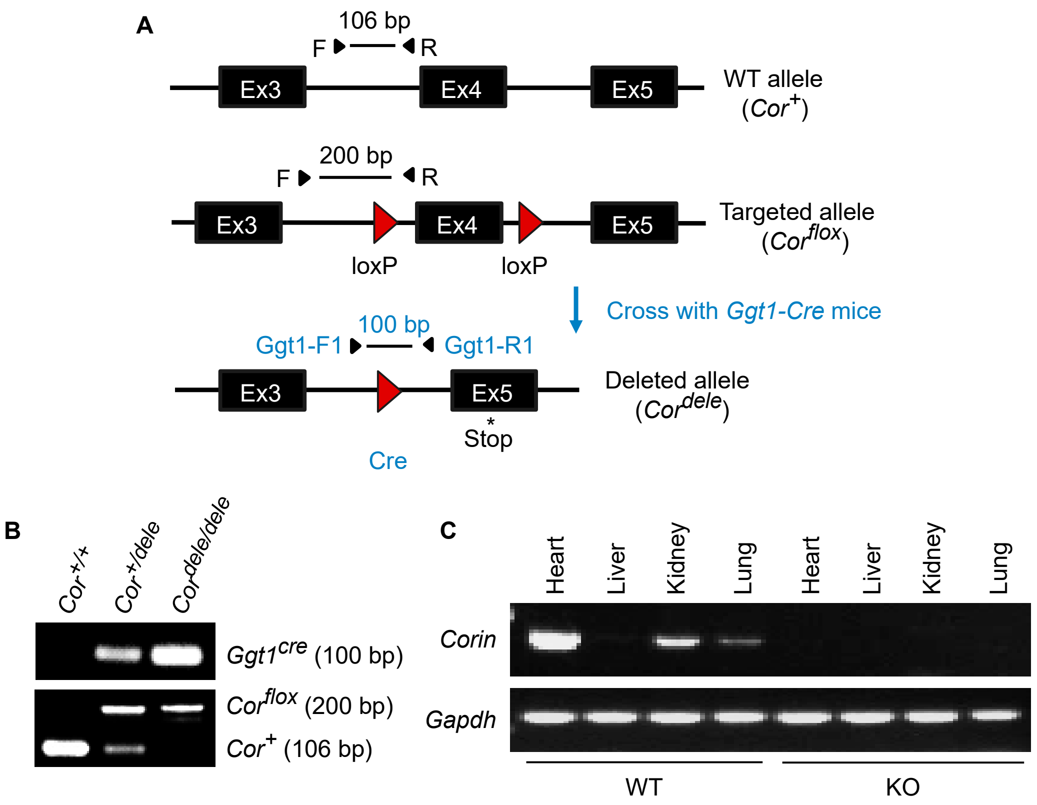

Figure S1. Generation of corin kcKO mice. (**A**) Schematic illustration of *Corin* WT allele (*Cor*+), *Cor*flox allele with two loxP loci flanking exon 4 in the *Corin* gene, and *Cor*dele allele generated by crossing *Cor*flox and *Ggt1-Cre* mice. Oligonucleotide primers used in PCR genotyping and expected sizes of PCR fragments are indicated. *Stop codon. (**B**) PCR genotyping in WT mice and heterozygous and homozygous mice with *Cor*dele allele. (**C**) RT-PCR analysis of *Corin* mRNA expression in tissues from WT mice and corin global KO mice generated by crossing *Cor*flox mice and *CMV-Cre* mice expressing *Cre* ubiquitously.

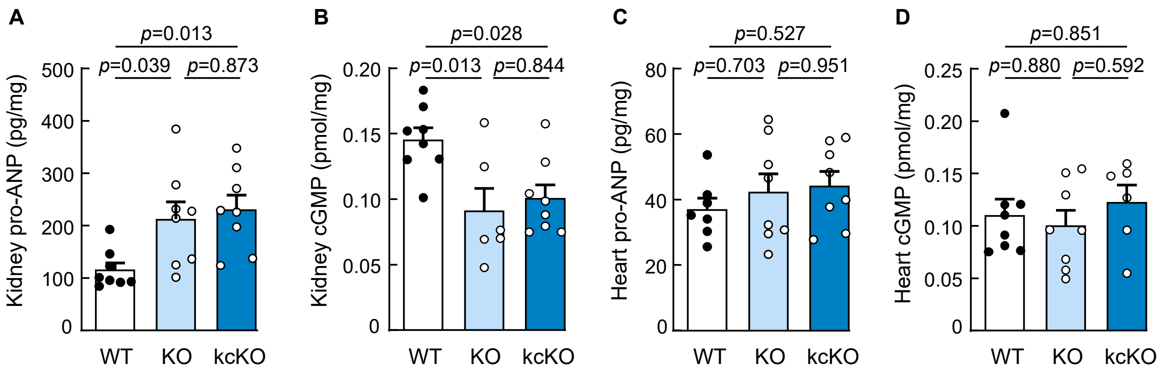

Figure S2. Pro-ANP and cGMP levels in the kidney and heart from female WT, corin KO, and kcKO mice. Kidney pro-ANP (**A**) and cGMP (**B**) and heart pro-ANP (**C**) and cGMP (**D**) levels in WT (black dots), corin KO and kcKO (white dots) mice. *n* = 6–8 per group. Data shown are mean ± SEM. *p* Values were analyzed by one-way ANOVA.

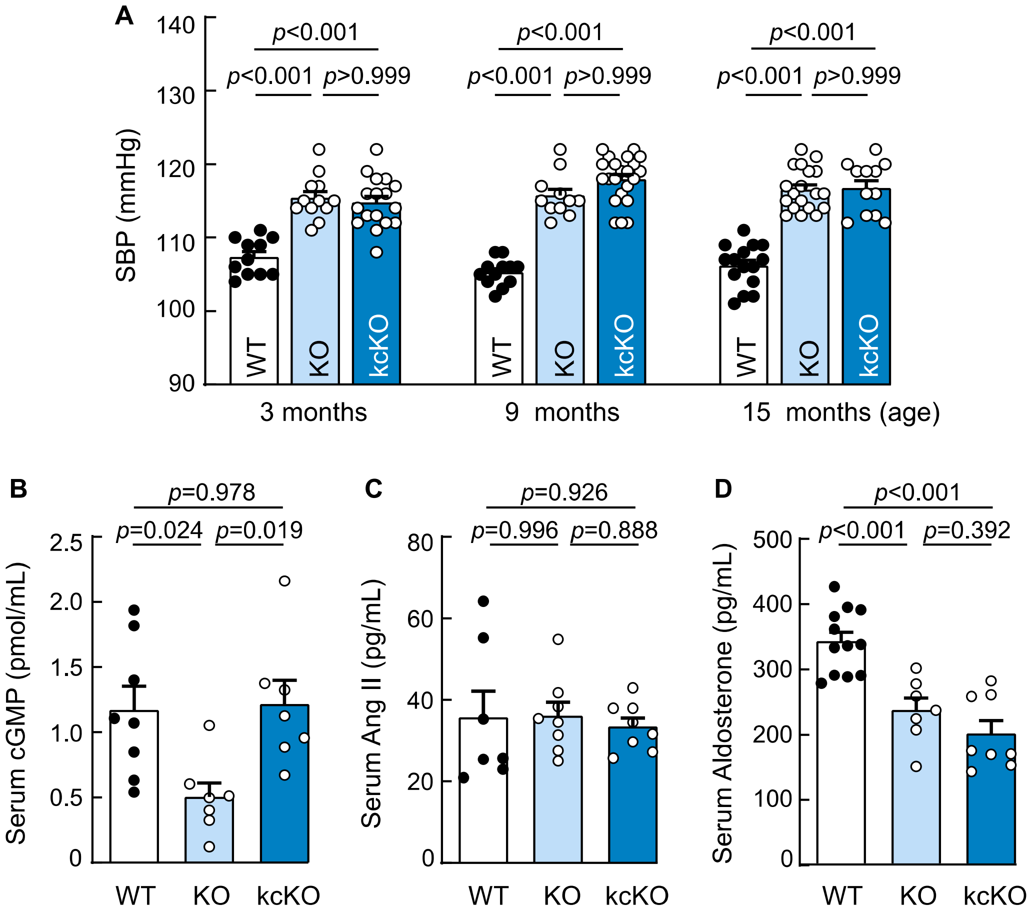

dots), corin KO, and kcKO (white dots) mice were measured by ELISA (**B**,**D**) or Enzyme Immunoassay (**C**). *n* = 7–12 per group. Data shown are mean ± SEM. *p* Values were analyzed by one-way ANOVA.

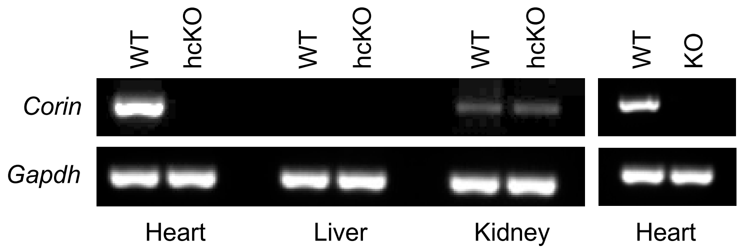

Figure S4. RT-PCR analysis of *Corin* mRNA expression in corin hcKO mice. *Corin* mRNA expression in the heart, liver (negative control), and kidney from WT, corin hcKO, and KO mice was examined by RT-PCR. *Gapdh* mRNA expression was used as a control. Data are representative of at least three experiments.

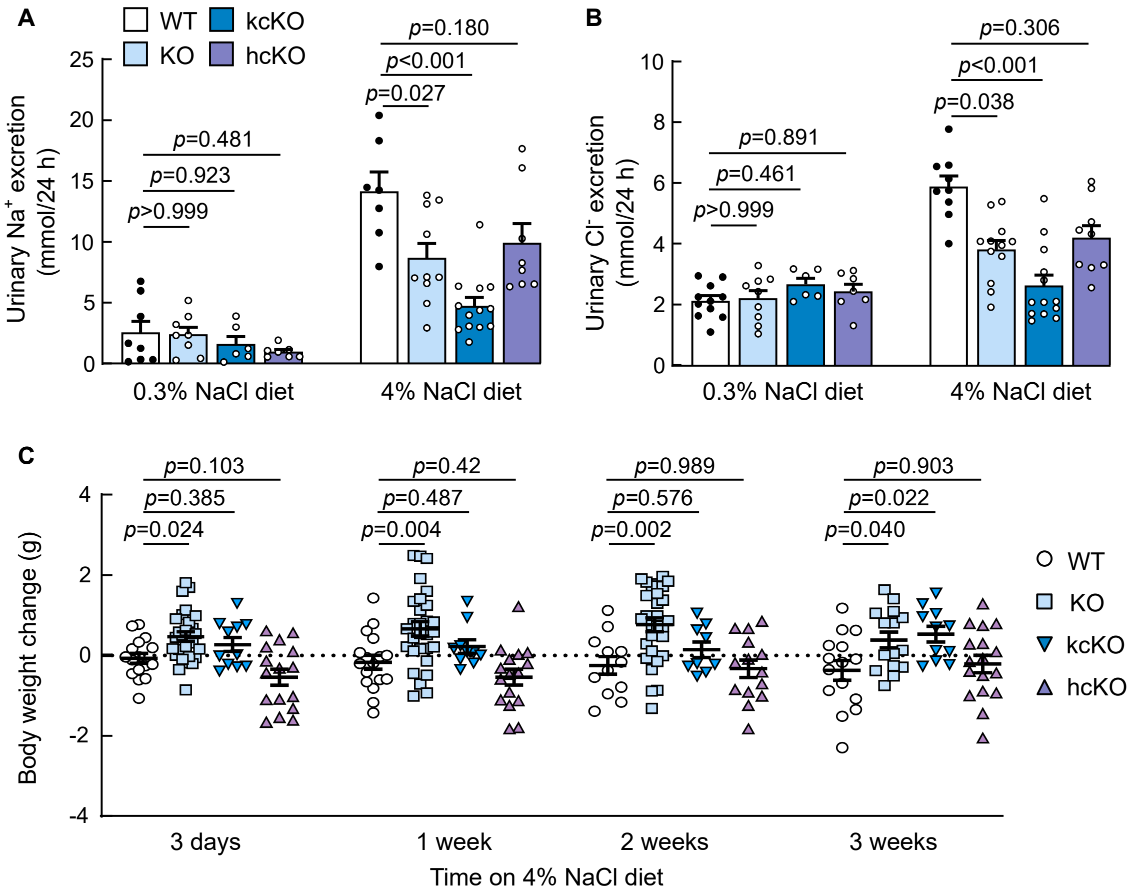

Figure S5. Urinary salt excretion and body weight change in female corin kcKO mice. (**A**,**B**) Urinary Na^+ (**A**) and Cl^- (**B**) excretion in female WT (black dots), corin KO, kcKO, and hcKO (white dots) mice on 0.3% and 4% NaCl diet were measured. *n* = 6–13 per group. (**C**) Body weight changes in female WT, corin KO, kcKO, and hcKO mice when 0.3% NaCl diet was switched to 4% NaCl diet. *n* = 9–29 per group. Data are mean ± SEM. *p* Values were analyzed by one-way ANOVA (**A**,**C**) or Kruskal-Wallis test (**B**).

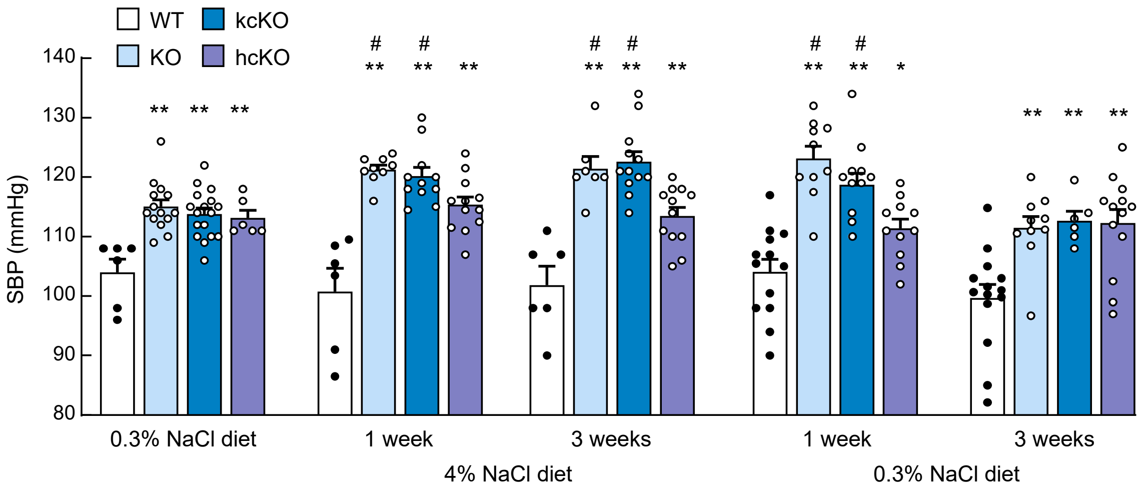

Figure S6. Salt-exacerbated hypertension in female corin KO and kcKO mice. Systolic blood pressure (SBP) was measured in female WT (black dots), corin KO, kcKO, and hcKO (white dots) mice on 0.3% NaCl diet, then 4% NaCl diet for one and three weeks, and back to 0.3% NaCl diet for one

and three weeks. * $p < 0.05$; ** $p < 0.01$ vs. WT of the same group. # $p < 0.05$ vs. the same genotype on initial 0.3% NaCl diet. $n = 6–17$ per group. Data shown are mean ± SEM. p Values were analyzed by one-way ANOVA.

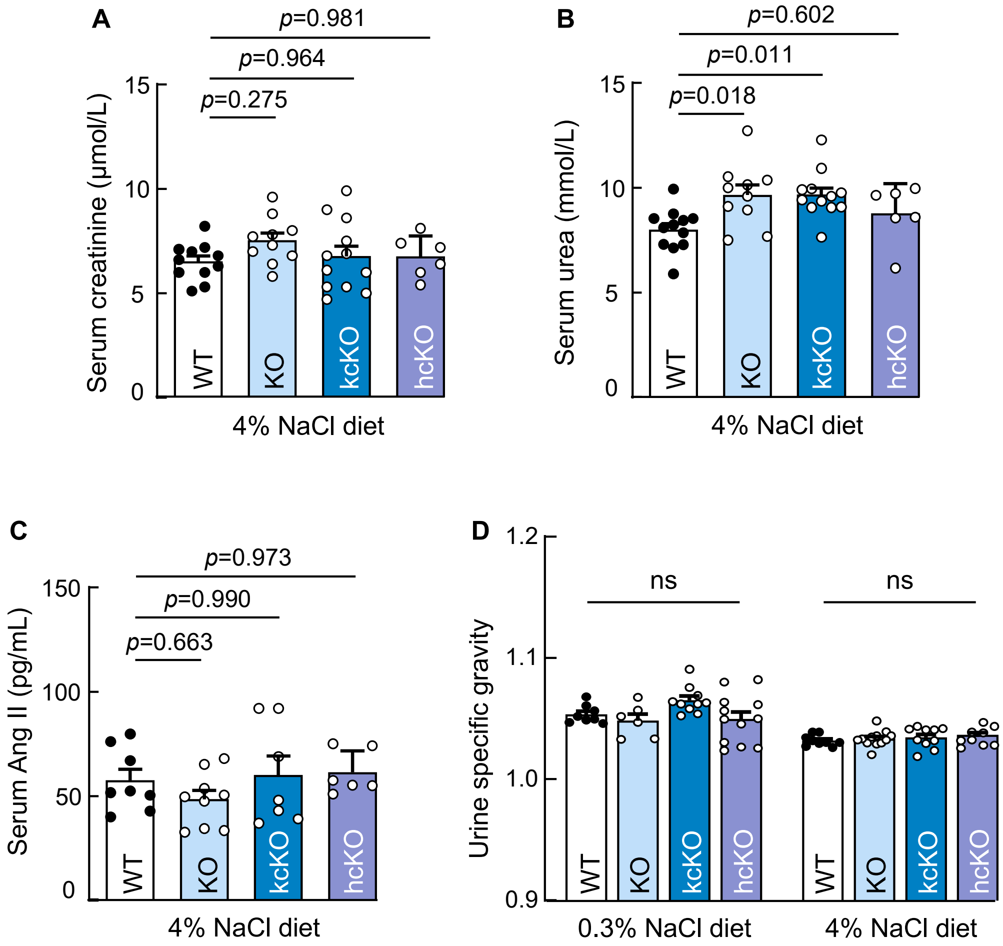

Figure S7. Serum creatinine and urea levels and urine specific gravity in male WT, corin KO, kcKO, and hcKO mice. (**A**–**C**) Serum creatinine (**A**), urea (**B**), and Ang II (**C**) levels in WT (black dots), corin KO, kcKO, and hcKO (white dots) mice on 4% NaCl diet were measured. $n = 6–12$ per group. (**D**) Urine specific gravity was measured in samples from WT, corin KO, kcKO, and hcKO mice on 0.3% NaCl and 4% NaCl diets. $n = 6–12$ per group. Data shown are mean ± SEM. p Values were analyzed by one-way ANOVA. ns, not significant vs. WT.

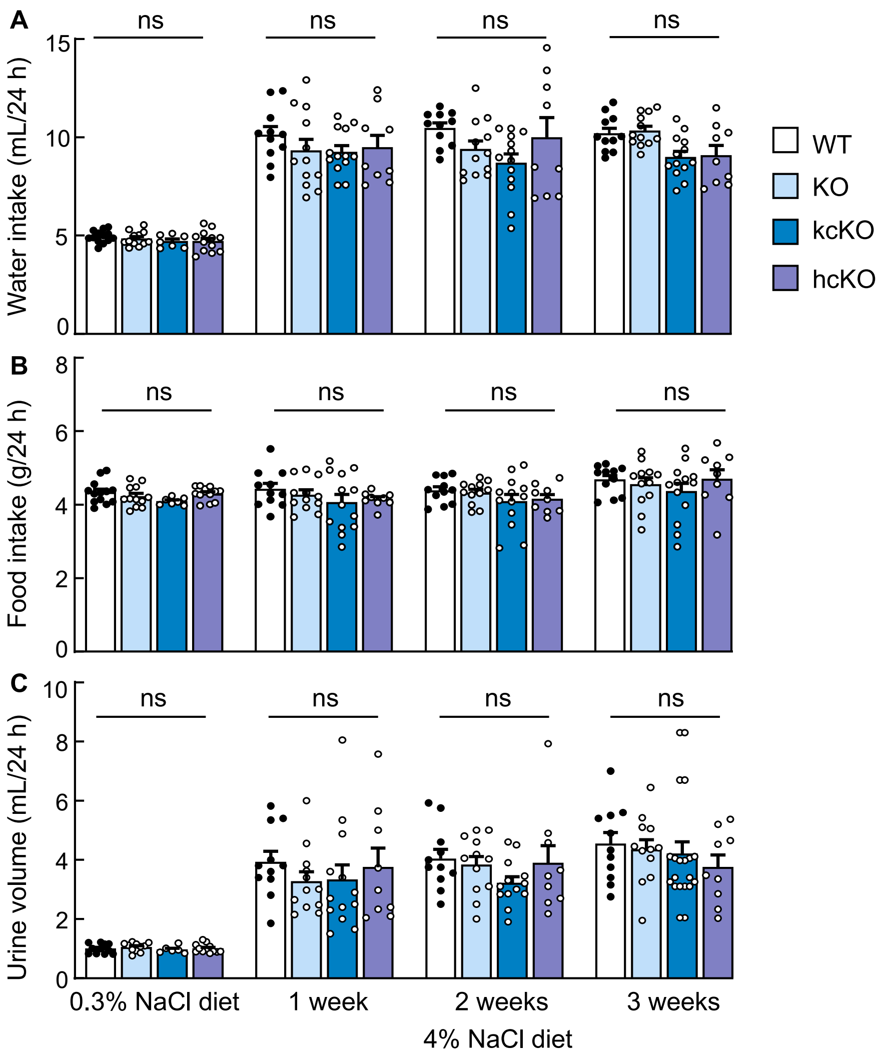

Figure S8. Water and food intakes and urine volume in female corin KO, kcKO, and hcKO mice. (**A**–**C**) Water (**A**) and food (**B**) intakes and urine volume (**C**) were measured in female WT (black dots), corin KO, kcKO, and hcKO (white dots) mice on 0.3% NaCl diet and 4% NaCl diet for one to three weeks. n = 6–20 per group. Data shown are mean ± SEM. p Values were analyzed by one-way ANOVA (**A**,**B**) or Kruskal-Wallis test (**C**). ns, not significant.

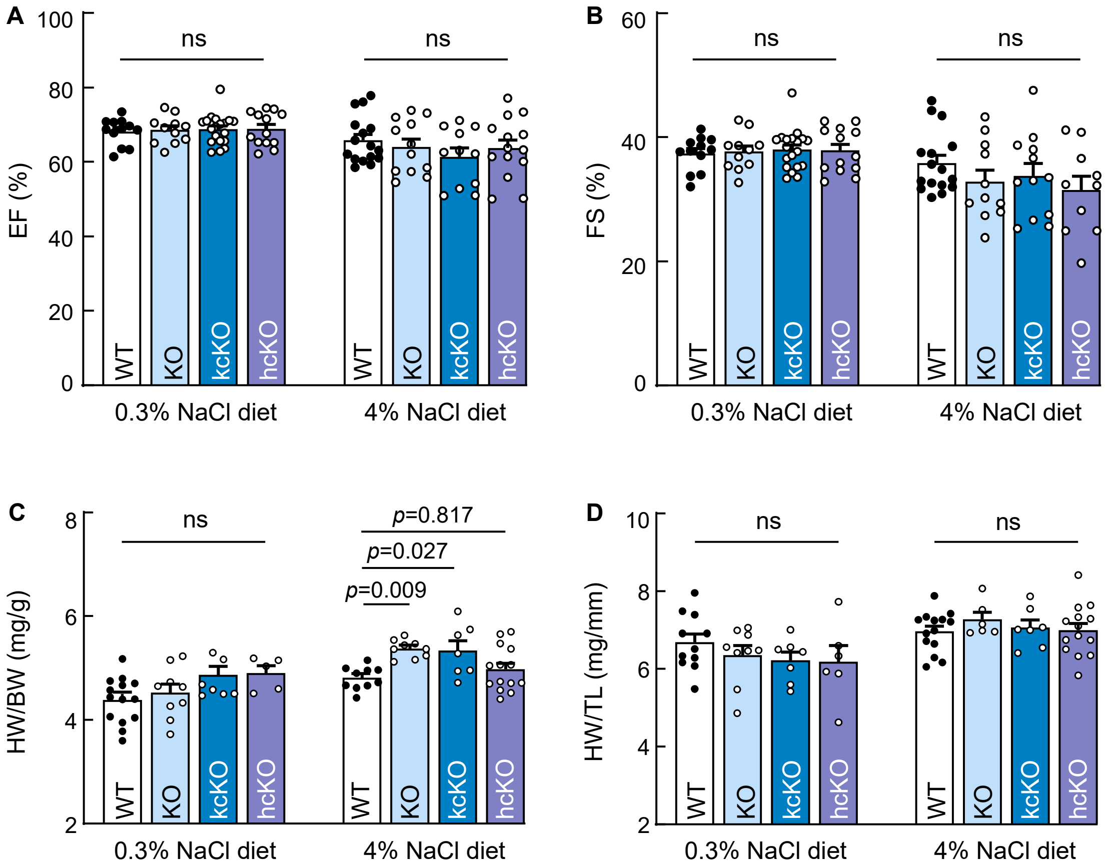

Figure S9. Cardiac hypertrophy in female corin KO and kcKO mice on 4% NaCl diet. (**A**,**B**) Cardiac function, indicated by ejection fraction (EF) (**A**) and fraction shortening (FS) (**B**), was measured by echocardiography in female WT, corin KO, kcKO, hcKO mice on 0.3% NaCl diet and 4% NaCl diet for three weeks. n = 10–19 per group. (**C**,**D**) Cardiac hypertrophy was assessed by ratios of heart weight (HW) to body weight (BW) (**C**) or tibia length (TL) (**D**) in female WT, corin KO, kcKO, hcKO mice on 0.3% NaCl diet and 4% NaCl diet for three weeks. n = 5–14 per group. Data shown are mean ± SEM. p Values were analyzed by one-way ANOVA. ns, not significant.

Table S1. Sequences of oligonucleotide primers used in PCR and RT-PCR.

Gene	Locus	Primer	Sequence	Size (bp)
Corin	NM_016869.3	forward	ATCACTCACAGCCAGTGTCAA	390
		reverse	TCCACAGAGTGATTGCTTTCCAT	
Corin	NM_016869.3	forward	TCAATTATGTGTGGCCTTCGGGGTG GTTTT	*Cor*$^{+}$ 106
		reverse	TCTATCTTATTGAAAAGTCTCTGTTT GT	*Cor*^{flox} 200
Gapdh	NM_001289726.1	forward	TGTTCCTACCCCCAATGTGT	138
		reverse	GGTCCTCAGTGTAGCCCAAG	
Ggt1	NZ_CP070291.1	forward	GCGGTCTGGCAGTAAAAACTATC	100
		reverse	GTGAAACAGCATTGCTGTCACTT	
